# Men’s experience of caring for a family member with cancer: a theory based on data

**DOI:** 10.1590/1518-8345.6679.4095

**Published:** 2024-01-26

**Authors:** Larissa de Carli Coppetti, Elisabeta Albertina Nietsche, Maria Denise Schimith, Cremilde Aparecida Trindade Radovanovic, Maria Ribeiro Lacerda, Nara Marilene Oliveira Girardon-Perlini

**Affiliations:** 1 Universidade Federal de Santa Maria, Santa Maria, RS, Brazil.; 2 Universidade Federal de Santa Maria, Departamento de Enfermagem, Santa Maria, RS, Brazil.; 3 Universidade Estadual de Maringá, Departamento de Enfermagem, Maringá, PR, Brazil.; 4 Universidade Federal do Paraná, Curitiba, PR, Brazil.; 5 Universidade Federal de Bahia, Salvador, BA, Brazil.

**Keywords:** Neoplasms, Caregivers, Men, Nursing, Grounded Theory, Symbolic Interactionism, Neoplasias, Cuidadores, Hombres, Enfermería, Teoría Fundamentada, Interaccionismo Simbólico, Neoplasias, Cuidadores, Homens, Enfermagem, Teoria Fundamentada, Interacionismo Simbólico

## Abstract

**Objective:**

to understand the meaning attributed by men to the experience of caring for their family member with cancer and to develop a substantive theory that represents the experience of men caring for their family member with cancer.

**Method:**

this is qualitative research guided by the methodological framework of Grounded Theory and the precepts of Symbolic Interactionism. A form with identification and interview data was used. The analysis followed the substantive and theoretical coding stages.

**Results:**

12 male caregivers of their family member with cancer participated. The constant comparative analysis of the data allowed the creation of a substantive theory “Experiencing the care of a family member with cancer: men as a caregivers” explaining the experience that has as its central category “The love that drives care”, representing the symbolic actions and attitudes of men living in the context of illness due to cancer and care

**Conclusion:**

the theory allowed us to understand feelings, perceptions, ways of acting and facing the diagnosis, providing care, recognizing difficulties and learning from the situations that arise, making explicit the interactional processes and symbolic elements present and how these influence male caregivers in their actions and attitudes.

## Resumen

**Objetivo:**

comprender el significado atribuido por los hombres a la experiencia de asistir a un familiar con cáncer y desarrollar una teoría sustantiva que represente la experiencia de los hombres acerca de dicho cuidado.

**Método:**

se trata de una investigación cualitativa guiada por el marco metodológico de la Teoría Fundamentada y los preceptos del Interaccionismo Simbólico. Se utilizó un formulario con datos de identificación y entrevista. El análisis siguió las etapas de codificación sustantiva y teórica.

**Resultados:**

participaron 12 hombres cuidadores de un familiar con cáncer. El constante análisis comparativo de los datos permitió elaborar una teoría sustantiva “Vivenciando el cuidado de un familiar con cáncer: el hombre como cuidador” explicando la experiencia que tiene como categoría central “El amor que impulsa el cuidado”, representando la acciones y actitudes simbólicas del hombre que vive en el contexto de enfermedad por cáncer y sus cuidados.

**Conclusión:**

la teoría permitió comprender los sentimientos, percepciones, formas de actuar y afrontar el diagnóstico, brindar cuidados, reconocer dificultades y aprender de las situaciones que se presentan, explicitando los procesos de interacción y elementos simbólicos presentes y cómo influyen en los hombres cuidadores en sus acciones y actitudes.

## Resumo

**Objetivo:**

apreender o significado atribuído pelos homens à vivência do cuidado ao seu familiar com câncer e elaborar uma teoria substantiva que represente a vivência de homens no cuidado ao seu familiar com câncer.

**Método:**

trata-se de uma pesquisa qualitativa norteada pelo referencial metodológico da Teoria Fundamentado nos Dados e pelos preceitos do Interacionismo Simbólico. Utilizouse formulário com dados de identificação e entrevista. A análise seguiu as etapas de codificação substantiva e teórica.

**Resultados:**

participaram 12 homens cuidadores de seu familiar com câncer. A análise comparativa constante dos dados permitiu a elaboração de uma teoria substantiva “Vivenciando o cuidado de um familiar com câncer: o homem como cuidador” explicativa da vivência que tem como categoria central “O amor que conduz o cuidar”, representando as ações e atitudes simbólicas do homem ao viver no contexto do adoecimento por câncer e do cuidado.

**Conclusão:**

a teoria permitiu conhecer os sentimentos, percepções, modos de agir e enfrentar o diagnóstico, desempenhar o cuidado, reconhecer as dificuldades e aprender com as situações que se apresentam, deixando explícito os processos interacionais e os elementos simbólicos presentes e como estes influenciam os homens cuidadores em suas ações e atitudes.

**HIGHLIGHTS:**

**(1)** For men, caring is a choice

**(2)** Taking care symbolizes a way of expressing love and reciprocation.

**(3)** Love in caring is strengthened by reciprocity, commitment, gratitude and zeal.

**(4)** To provide care, man reorganizes himself and adjusts to the conditions that present themselves

**(5)** Male caregivers need to be heard and included in the actions of health teams.

## Introduction

Currently, cancer is one of the most critical health problems in the world, given its high incidence and magnitude ^(^ 1 ^)^. Evidence shows that the disease can reduce people’s ability to perform self-care, making them face numerous limitations ^(^ 2 ^)^. In this way, receiving a diagnosis of cancer not only has an impact on the person affected, but also affects the entire context of their family, thus, being considered a family disease, whether from a genetic point of view that may be present, as well as the disorder that causes in the daily structure of the family ^(^ 3 ^)^. 

The individual who becomes ill feels the need to be cared for, understood, loved and, above all, to have someone to share their fears and anxieties. Faced with this, the family reorganizes itself and makes its own time more flexible in order to create strategies to deal with adversities, dedicating time and energy to care, in the new routine established by the illness ^(^ 4 ^)^. In this process, the family member is chosen who will be primarily responsible for the care demands that emerge with the disease, considered the main caregiver. 

Studies affirm the majority presence of women in the role of main caregivers when faced with cancer ^(^ 2 ^,^ 5 ^,^ 6 ^-^ 7 ^)^, reinforcing the historical and cultural issues associated to women the maternal and welcoming profile, whereas men are usually associated to the role of family health providers ^(^ 8 ^)^. However, in the current context, this pattern has been undergoing changes, highlighting the insertion of men in the position of caregiver ^(^ 9 ^,^ 10 ^-^ 11 ^)^, given the increasing inclusion of women in other social roles, including formal work ^(^ 9 ^)^. 

When studying couples in which one of the partners needs care, research reinforces these perspectives worldwide, indicating that men’s response to the need for care for their spouses was similar to that of women, which also allowed us to infer that men assume the responsibility role of caregiver required in systems that provide care at public and private levels in Germany ^(^ 12 ^)^. 

It is known that providing care is not an easy task for any caregiver, especially when a family members becomes ill with cancer, given the stigma and high mortality rates associated with the disease ^(^ 5 ^)^. For men, caring can be even more challenging, considering the lack of preparation for such activities, which can put them in a situation of constriction and interfere with the way they experience care. Therefore, the considerations of care as something related to the feminine universe, socially and culturally constructed over the years, need to be considered nowadays, ceasing to look at care only from the female perspective, but rather, understanding how care takes place in the male dominance. 

The literature is considered extensive regarding the study of caregivers, however, it is clear that researches addressing male caregivers is still scarce. A study with the objective of analyzing the trend of scientific productions in Brazilian postgraduate programs regarding the experience of men as responsible for care highlighted the reduced number of scientific productions on the subject, indicating a gap in scientific knowledge and the progressive need to study the demands of the male population in different contexts, especially in care ^(^ 13 ^)^. 

Caring is a dynamic process that continually changes based on the meanings resulting from the caregiver’s interaction with themselves, with the sick person and with the environment. Based on these considerations, it is assumed that men, when faced with cancer and the need for care, define the situation based on past experiences, perspectives for the future and attributed meanings ^(^ 14 ^)^. 

Therefore, this study aims to: understand the meaning attributed by men to the experience of caring for their family member with cancer and develop a substantive theory that represents the experience of men in caring for their family member with cancer.

## Method

### Study design

This is a qualitative research guided by the methodological framework of Grounded Theory (GT) ^(^ 15 ^)^, which proposes the development of a theory based on data obtained and analyzed in a systematic and comparative way ^(^ 15 ^-^ 16 ^)^, and as a theoretical framework, Symbolic Interactionism (SI) ^(^ 14 ^)^, which has a perspective of analyzing human experiences with a focus on interactions ^(^ 14 ^)^. 

### Setting

Participants were recruited in two hospitals located in two cities located in the state of Rio Grande do Sul/RS, Brazil. The choice for these two different services was due to the characteristics presented, one being a public institution and the other a private one, which allows access to participants from different sociodemographic contexts.

### Period

The interviews were carried out from January 2020 to October 2021.

### Participants and selection criteria

The study participants were men who were the main caregivers of a family member with cancer. The search began with the recognition of patients diagnosed with cancer receiving care in the selected sectors, when a first face-to-face contact was made, made by the researcher or the scientific initiation scholarship member participating in the research, to identify their main caregiver, and if this met the following inclusion criteria: being a man who performs primary caregiver activities, that is, the family member with all or most of the responsibility for care, and being 18 years old or older. Men were invited to join the study through face-to-face conversation or telephone contact. Caregivers who had cognitive limitations that prevented them from understanding or answering the interview questions, evident in the initial approach, were excluded from the study.

The selection of caregivers, the number of participants and the formation of sample groups were defined following theoretical sampling, as recommended by GT. As a result, men were progressively selected to take part in the study, until the moment when theoretical saturation was reached, that is, no new dimensions or relationships emerged during the analysis that would contribute to the understanding of the categories and formulation of the theory ^(^ 16 ^)^. 

Initially, data collection was carried out with men who cared for their family member during the hospital stay, who made up the first sample group. It is noteworthy that this condition was not defined intentionally, but rather according to the availability of the first participants. During the interviews and concomitant data analysis, coding the information and comparing it, using the concept of data circularity, questions arose regarding the experience of the man who provides care in another context, that is, outside the hospital environment. Therefore, the collection was directed to caregivers of patients undergoing outpatient treatment, in which care involves actions at home and monitoring for treatment, constituting the second sample group.

### Data collection and analysis

Data collection occurred through interviews conducted by a single researcher, nurse, doctoral student, with experience in the hospital area and with the presence only of the male caregiver. Information collection began with data to identify and characterize the caregiver such as age, education, marital status, profession, relationship with the caregiver, help with care and previous care experience; and their sick family member information on gender, age, diagnosis and time since diagnosis, according to an instrument prepared by the research team. Afterwards, the interview itself began, with the guiding question: tell me what it is like for you to care for your family member with cancer? This was the question that defined the researchers’ interest, and as information emerged, other questions were raised, aiming to expand verbalization and encourage participants to articulate their own intentions and meanings resulting from their experience, allowing for a deeper understanding of the researched phenomenon. An interview was carried out as a pilot test to confirm the applicability of the questions and familiarize the interviewer with the collection process, which was subsequently included in the data analysis due to the quality of the information obtained.

The data were analyzed according to the precepts of GT simultaneously with data collection, by the same researcher responsible for the interviews, using constant comparison procedures, inductive-deductive reasoning, data circularity, theoretical sensitivity, memos and diagrams ^(^ 15 ^)^. 

In this study, coding, as data analysis is defined in GT, followed the steps of substantive coding (open and selective) and theoretical coding ^(^ 15 ^)^. In open coding, the interviews were explored in detail, broken down line by line, with a view to identifying incidents capable of demonstrating a pattern of man’s behavior in his experience as a caregiver. At that time, we sought to identify what each incident represented, what it wanted to show, what it meant and, based on this, a name was given, called a code. 

With the creation of the coding tree, the codes were compared with each other and grouped by similarities and differences, adding them to others that expressed the same type of behavior, forming groups. The preliminary groups or categories received more abstract conceptual names than the codes and, as the interviews were carried out and coded, the new codes were inserted into the categories already created or formed new categories. Therefore, the themes presented were derived from the data.

During the analysis, the central category was identified, characterized as a complete and potentially explanatory concept, through which the other categories are unified and establish relationships. This was identified by the frequency with which it appears in the data, being able to expose the meaning of the action in the studied context, and together with the other categories, express how the research subjects experience the problem in question ^(^ 16 ^)^. 

Following the substantive coding and identification of the central category, theoretical coding began, which highlights how the categories relate to each other and make up the substantive theory. Theoretical coding was carried out based on the theoretical code of the six Cs, capable of representing the interrelationships found in the categories through contexts, causes, contingencies, intervening conditions and consequences ^(^ 16 ^)^. 

As part of the data analysis process, diagrams and memos were created that contributed to understanding the interactions between categories, subcategories and codes. Furthermore, these can be important tools for identifying the central category and the substantive theory representative of the phenomenon under study.

In order to assist in organizing the data, the Nvivo 11 ^®^ software was used, a mixed and qualitative analysis tool, which allows the researcher to organize the content of interviews, facilitating the analysis and coding of data, based on the conception and the initial sampling procedures, theoretical development and presentation of results. 

The validation of the substantive theory and the elaborated diagram was carried out with experts in conducting studies using GT during group meetings, which helped with the necessary adjustments to the final diagram of the theory, allowing it to reveal how the experience of male caregivers happens. of your family member with cancer.

### Ethical aspects

The study respected ethical principles, having approval from the Ethics and Research Committee under the number 4,223,230. All participants agreed to participate in the study voluntarily and signed the Informed Consent Form. The anonymity of participants was guaranteed, and they were identified by the letter C (caregiver) followed by a numerical sequence in the order in which they took part in the research.

### Rigor

The credibility of the study is due to the data analysis carried out carefully by the researcher, as well as the presentation of the narratives that elucidate the results ^(^ 17 ^-^ 18 ^)^. The description of the participants’ characteristics provides transferability, the guidelines from the Consolidated Criteria for Reporting Qualitative Research (COREQ) ^(^ 19 ^)^ guide provide reliability, and the indication of limitations and positive aspects, as well as the researchers’ reflexivity, reveal the confirmability of the study ^(^ 17 ^-^ 18 ^)^. 

## Results

The research involved the participation of 12 male caregivers aged between 27 and 75 years, four children and eight spouses of the family member who was ill with cancer. Seven of them were not working at the time of the interview, eight reported having experience with caring for family and friends previously and 11 had the support of family and friends for care. The age of the sick person ranged from 29 to 77 years old, two were men and ten were women. The initial diagnoses involved breast, uterine, colon, pancreas, kidney and lung cancer, and most of them had metastases to other organs.

Of all the men identified as main caregivers who met the research criteria, one of them did not return contact, and one agreed to participate in the study in the first approach, but subsequently did not return attempts to schedule the interview.

Six of the interviews were carried out in person, in a room provided by the institution for recruiting caregivers, and, considering the pandemic period that took place during data collection, to enable the continuity of the study, six interviews took place virtually, by video call via the Google Meet app or video call via the WhatsApp app. The interviews lasted an average of 1h30 minutes and were audio recorded.

The male caregivers were divided into two sample groups, six belonging to the first group who provided care in the hospital environment and six provided care in outpatient treatment and at home.

Data analysis, following the methodological steps recommended by GT, from the perspective of IS, allowed understanding the experience of male caregivers of their family member with cancer, as well as the pattern of behavior and the meanings attributed by them. The concepts and their interrelationships identified in the data analysis belong to an analytical process that exposes the definitions, actions and decisions of men from the moment they receive their family member’s cancer diagnosis until the performance of care.

From the coding and analysis process, four concepts were organized – “(Re)Discovering the cancer diagnosis”, “Adjusting to the reality that presents itself”, “Being guided by care” and “Noticing changes resulting from care”. These concepts integrate and support the central concept “The love that drives care” which makes it possible to identify the relationships that are established between the categories and makes up the experience of men when caring for their family member with cancer.

**Figure 1 -:**
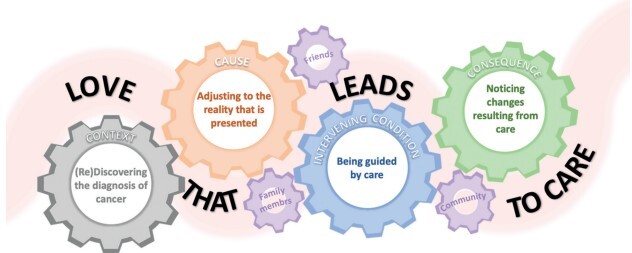
Representative diagram of the substantive theory: experiencing care for a family member with cancer: the man as caregiver

The relationship between the central concept and the other concepts is made clear in Figure 1, which presents the substantive theory: “Experiencing the care of a family member with cancer: the man as caregiver”. The substantive theory produced from the phenomenon studied highlights the concepts and their properties, demonstrating the path taken by man in his experience as a caregiver, based on the relationships of four theoretical codes: context, cause, intervening condition and consequences ^(^ 16 ^)^. 

“(Re)Discovering the diagnosis of cancer” is the context in which the man’s experience occurs, permeated by the repercussions of discovering his family member’s illness. The feelings that emerge when communicating about the family member’s illness include negative and suffering aspects, which are intensified by the significance of cancer based on painful treatments, difficulty in healing and the finiteness of life. This moment is impactful for male caregivers, which makes them feel lost and helpless, as illustrated in the statement: *When we received her cancer diagnosis, it was the same thing as taking me off my feet, there was a hole left* (C6). 

Having your life and that of your family member impacted by cancer brings to the male caregiver the perception of living in an unknown world. Cancer is considered obscure, because, even if the caregiver has preconceptions and established meanings for the disease throughout their life, the experience of participating in the illness up close, as someone who is responsible for caring, is often new, as expressed in the excerpt from the interview: *At first I was pretty shaken, I was terrified, I think it’s normal, because we don’t know the disease* (C2). 

The man is faced with something new, different from his experience up until now, and with this he mobilizes himself to adapt to the conditions and functions imposed by the illness of his family member. “Adjusting to the reality that presents itself” reveals the cause of the phenomenon studied. The man takes care, therefore, adjusts to the demands that arise when his family member becomes ill, developing necessary actions and decisions to include care in his life.

Realizing the need for care makes caregivers begin the process of reflection and reframing the situation, adopting actions compatible with the imposed reality. Being the main caregiver for your family member with cancer is the result of this process of interaction that occurs in the face of the unknown, and guided by the love that evolves the relationship, they decide to care. *I take care of her because I wanted to, it was my choice. It’s a way of trying to convey all the love I feel. For love, always for love* (C3). 

Care expressed as an option, gratitude, commitment and wanting to be close are all linked to feelings of affection and affection, representing ways of demonstrating love, which in experience is the central element that guides choices and the desire to take care of your loved one. familiar. With this new attribution, the man mobilizes himself to recognize the symbolic elements present in this reality, seeking to interpret the needs and move towards the resources required for care.

Changing habits, readjusting roles and priorities is part of the process of including yourself and adjusting to the new assignment. To care for their family member and meet the demands of cancer treatment, which is generally long and complex, the caregiver’s decisions aim to create availability to provide care within their life context. *I stopped working to be with her, for whatever she needs. So, I don’t know if I’ll go back to work, I think I’ll just take care of her!* (C4). 

Taking into account the time of treatment and the care demands, the caregiver, within his organization and seeking to understand what the situation requires, starts to request help, so that people can, in some way, help him at that moment. This support is sought by men from people who inspire confidence in them, such as family, friends and members of the community, creating bonds of partnership that are capable of strengthening them in the daily fight against cancer. *If I hadn’t had my parents’ support at the beginning, I don’t know how I would have done it (C1). There are friends that we don’t even have to thank, a big thank you is not enough. Thanks to them I can be here* (C6). 

The mental process instituted taking into account the conditions of illness and care, and which provides new definitions and learning, contributes to man moving forward. “Being guided by care” presents itself as the intervening condition, demonstrating actions and attitudes that can, in some way, modify the feelings, perceptions and meanings related to the experience.

In addition to the social interactions that modulate actions, there is the importance of the self, as although it is also determined by interactions with others, it portrays the individual’s internal environment. Therefore, recognizing feelings, attitudes carried out in the experience and the difficulties that arise, are characterized as conditions that can intervene and/or modify the phenomenon studied.

Accompanying the beginning of cancer treatment provides new sensations and meanings related to the outcome of the disease, helping men to increase their faith and hope for a cure, as stated in the statement: *We know it’s difficult, it’s a complicated disease, but when there’s still a treatment option, that helps us look forward* (C12). 

The performance of care is conducted according to the needs of the family member and the man begins to perceive himself in the position of caregiver. In this process, they create strategies so that the negative feelings and weakness they feel are not noticed by their family member, maintaining strength and positivity in the face of treatment. *I try not to show any weakness. I try to appear as strong as possible, giving her strength. Because it is a difficult time, she deserves all the love and affection in the world* (C10). 

When traveling along the path of care, one recognizes some difficulties related to the conditions of being a man who needs to move from one position to another – being cared for in order to be a caregiver. Despite mentioning their love and desire to be caregivers, the unexplored environment of care brings some challenges, reiterating the historical and social trends that place women as the main figure responsible for caring within the family space. Difficulties related to gender and lack of skills are noticed at the beginning of the experience and are gradually given new meaning and addressed.

*The lack of instinct that every woman seems to be born with, to take care of others. As a man, I think I wasn’t born with this instinct, it was very difficult at the beginning. Nowadays I’ve gotten used to it, I’ve gotten used to it, but it’s very strange*(C1) *. There were several difficulties, because I had never applied a dressing, looked after a drain, given a bath, I had to learn how to do everything, wash clothes, make food* (C3). 

The changes brought about in the life of the person who becomes ill make the man face the difficulty of living with suffering due to the repercussions of the disease. Knowing how to deal with changes in the physical and emotional conditions of your family member is an inherent role in the role of the caregiver, which is why they need to seek resources and ways to face the adversities that are present in the experience of care. *The difficulty is that I feel very sorry for the fact that she had side effects from the surgery, it sometimes hurts to hear her say: “I can’t wear jeans anymore, they are too tight and the bags appear”. I feel sorry for her, for her having to change her life* (C4). 

The way in which man mobilizes and develops the ability to respond to problems that arise during care, using individual and collective resources based on a reflective and analytical mental process, is what promotes as a consequence of the experience - “Noticing changes arising of care.” Consequences result from actions and interactions of variable characteristics, some individual and others collective. Following the premises of SI, individuals carry out interpretative processes considering, in addition to themselves and their self, the actions and social interactions with others and the environment in which they experience care and, as a result, they are able to perceive the consequences of being caregivers.

Experiencing care with all the uncertainties, anxieties, fears and challenges, provides changes in the way of thinking and acting. With this ability to perceive, the experience of following the illness and caring for your family member is no longer associated only with negative feelings and difficult situations. The reframing of what has been experienced allows us to perceive events as potentializing a transformation in the understanding of facts and ways of living with other people. Therefore, even when faced with the impact of the illness of a family member, male caregivers recognize that cancer has changed the way they think and act in the face of various life situations.

*I learned to value everything I have, I learned to live in the moment, not worry about tomorrow, what I will acquire tomorrow. Live what we live today. The best, because we have to live life one day at a time. Life has a great price, a great value. So, people would give up, we earn money, we earn our dreams, I didn’t bury my dreams, I have them all here, they may be delayed one day or another, but I’ll go looking for them at the right time. Today, the moment we live in, is the time to take care of our partner, to be a companion. Because the person we love we have to show it in actions, not just in words* (C2). 

Enjoying the moment, the company of the family member and believing that everything has a purpose, makes men continue living beyond the illness, nourishing the love and gratitude of being able to care for their family member.

## Discussion

The results identified, based on the understanding of the experience of the male caregiver, reinforce that receiving the diagnosis of oncological disease was impactful, causing great suffering and emotional destabilization. Doubts and uncertainties emerge in the face of the need to deal with the unexplored world of illness and care. The literature shows, when studying gender differences in the burden of caregivers for aging partners, that caring for women is characterized as an extension of a social role, whereas for men it constitutes a new and unknown attribution ^(^ 20 ^)^. 

New situations allow the elaboration of new symbolic meanings, however, illness related to cancer brings back several universal meanings, considering the definitions of an invasive, aggressive disease that is difficult to manage and brings people closer to death. Therefore, cancer presents itself as a setback in the lives of family members and caregivers, causing alignments of individual actions, so that, in a cooperative way, everyone acts in search of a resolution to the problem ^(^ 14 ^,^ 21 ^)^. From an interactionist understanding, it can be understood that the way people act in the face of a given situation results from shared symbolic interaction, so that the interpretation and definition of the moment in which they are living lead to a perspective that is common ^(^ 14 ^,^ 21 ^)^. 

That said, it is understood that care is produced based on the senses and symbolic meanings attributed and shared in view of the needs of those who are cared for. As a result, the family caregiver adjusts according to the way they perceive the illness situation, which varies as interactions occur with society, the environment and the ill person ^(^ 14 ^)^. 

In the experience of the male caregivers in this research, caring was a choice, marked by the feeling of love that is strengthened through retribution, gratitude and commitment. The literature describes that men demonstrate moral motivations and social obligation to care, and the retribution of parental care received is an important factor for the male child to decide to care for his dependent father/mother ^(^ 11 ^)^. For spouses, caring represents a responsibility established in the marriage act, and develops a relationship based on respect, patience, trust and love ^(^ 22 ^)^. Caregivers feel called to care given the need that arises in the family, emphasizing that motivation occurs through love and duty within the family dynamics ^(^ 23 ^)^. 

The gift theory presents an interpretative model for thinking about the foundations of solidarity and alliance in society, based on the permanent presence of a set of reciprocities of an interpersonal nature. Reciprocity presupposes concern for the other, having as its essence the triple obligation of “giving, receiving, reciprocating” ^(^ 24 ^)^. Reflecting on this concept, a relationship can be established between the gift theory and reciprocity in the practice of care. It is clear that the man has the gift, he gives himself to take care of his family member, and reciprocity is part of the process of assuming care as a possibility of being able to reward behaviors already received. A family member who becomes ill, be it a spouse, father and/or mother, is cared for because at some point in their life they were cared for. Likewise, it is understood that the act of caring transposes the desire to someday be able to be reciprocated, if today I care, I will be able to be cared for in the future. 

The behaviors that favor the performance of care are products of the reflective and interpretative process that each individual establishes with themselves, using the action of the mind and the self. Furthermore, the ability to place oneself in others provides the definition of their behavior based on what they consider to be the best offer of care. For SI, putting oneself in someone else’s shoes is considered an important mental activity, essential to symbolic communication and self-development ^(^ 14 ^)^. This allows the individual to act morally, have empathy, help, protect and control their own actions, realizing the consequences of these for their life and that of their family. 

From this perspective, despite the impact that cancer has on the lives of caregivers, they place the needs of their family member above their own. Even though care is considered something new, they use love, perceptions and the desire for retribution to accompany and be present during cancer treatment.

Care is an important value for man, and becomes willpower, as well as a source of strength to overcome the adversities that present themselves and assign new definitions and perceptions about life, whether in the present moment – what they are living today, in the past – what they have already lived, or in the future – what they still have to live. Thus, it is possible to understand care as a human action that arises from the actions-interactions between those involved and the caregiver’s self-interaction ^(^ 25 ^)^. 

Resilient attitudes are related to the development of skills capable of responding positively to obstacles that arise, which can be seen in the experience of male caregivers. By demonstrating positive attitudes and definitions towards the reality they find themselves in, they are able to give new meaning and identify new ways of living life. These actions and reactions are explained by the self-interaction that the individual establishes to guide their conduct through their self, according to the premises of SI ^(^ 14 ^)^. 

From this perspective, men’s experience of care is something complex that demands adjustments. By studying them, providing listening, attention and support, one can contribute to strengthening positive and resilient attitudes, and negative feelings of fear, uncertainty and anguish can be minimized, providing the opportunity for the reality of care to be effectively conducted in a calmer manner.

The evidence highlighted in this study contributes to the care praxis of nurses who care for family caregivers. By knowing the experience of male caregivers and caring for them from this perspective, it allows us to reflect on the needs focused on what makes sense to them, and thus, direct actions and care strategies that, in some way, can meet what they need. Furthermore, providing care to male caregivers will help them to be seen as individuals who care and also deserve attention, support and assistance.

Furthermore, it is pointed out that knowing the gender characteristics that exist in informal care, as well as their impact on the health and quality of life of caregivers, must be incorporated into nursing assessments and interventions so that, in an individualized way, they promote improvements in health of caregivers according to their gender ^(^ 26 ^)^. 

As limitations of the study, the global pandemic created by the coronavirus (COVID-19) stands out, which has placed everyone in a situation of social isolation, making it necessary to outline strategies capable of allowing the research to progress, using telephone contact and online interviews. It is known that this modality, despite being able to capture the statements, emotions and feelings of caregivers, does not allow for a relationship of closeness and complete attention, as occurs in face-to-face interviews.

## Conclusion

The discovery of a family member’s cancer, from the perspective of male caregivers, is shocking and full of negative feelings and uncertainties related to the future. Taking on care is a choice and symbolizes the way of expressing love and reciprocating the care already received. The interactions and definitions of events direct the way of acting and determine the symbolic meanings attributed to the experience of being a man who takes care of a sick family member. From this perspective, it is understood that caring for a family member with cancer, in the experience of male caregivers, has the symbolic meaning of love that is strengthened by reciprocity, commitment, gratitude and care.

The results achieved allow us to see that there is still a lot to study and deepen our knowledge about male caregivers. However, the substantive theory developed provides impacts for research, practice and extension, as it presents indications for ways to serve the male public who provide care and, also, brings propositions for possible studies on the subject, which present broad possibilities.

The study also reinforces the importance of encouraging nursing students, from the beginning of their training, to develop attentive listening in order to identify the singularities and needs of male caregivers, in order to understand what this experience means for them, building indications of care from that look.
